# Supplementary material for: A UMLS-based spell checker for natural language processing in vaccine safety
Source: BMC Med Inform Decis Mak. 2007 Feb 12;7:3. doi: 10.1186/1472-6947-7-3 (PMC1805499; doi:10.1186/1472-6947-7-3)
Supplement: Additional file 13 — RAP application directory. Sets up directory for RAP (RDF application for PHP) source code [file 1472-6947-7-3-S13.gz › rap/doc/phpdoc/resModel/ResLiteral.html]

xml version="1.0" encoding="iso-8859-1"?


Docs For Class ResLiteral


## Class ResLiteral

Description

Description |
Methods (details)

An RDF literal.

The literal supports the xml:lang and rdf:datatype property. For XML datatypes see: http://www.w3.org/TR/xmlschema-2/

History:

- access: public
- author: Daniel Westphal <at d-westphal dot de mail at d-westphal dot de>
- version: V0.9.1

Located in /resModel/ResLiteral.php (line 23)

```
Object
   |
   --Node
      |
      --Literal
         |
         --ResLiteral
```

Method Summary

Description |
Methods (details)

ResLiteral
ResLiteral
(string $str, [string $language = null])

object model
&getAssociatedModel
()

void
setAssociatedModel
(object model &$model)

Methods

Description |
Methods (details)

Constructor ResLiteral (line 40)

Constructor You have to supply a string.

ResLiteral

ResLiteral
(string $str, [string $language = null])

- string
  $str: label of the literal
- string
  $language: optional language identifier

getAssociatedModel (line 63)

Get the reference to the assocoated model.

- access: public

object model

&getAssociatedModel
()

setAssociatedModel (line 52)

Sets the reference to the assocoated model.

- access: public

void

setAssociatedModel
(object model &$model)

- object model
  $model

#### Inherited Methods


Inherited From Literal

> Literal::Literal()  
> Literal::equals()  
> Literal::getDatatype()  
> Literal::getLabel()  
> Literal::getLanguage()  
> Literal::setDatatype()  
> Literal::setLanguage()  
> Literal::toString()

Inherited From Object

> Object::toString()

Documentation generated on Thu, 7 Jul 2005 13:43:38 +0200 by phpDocumentor 1.3.0RC3
